# Prevalence of Musculoskeletal Post-COVID Pain in Hospitalized COVID-19 Survivors Depending on Infection with the Historical, Alpha or Delta SARS-CoV-2 Variant

**DOI:** 10.3390/biomedicines10081951

**Published:** 2022-08-11

**Authors:** César Fernández-de-las-Peñas, Ignacio Cancela-Cilleruelo, Paloma Moro-López-Menchero, Jorge Rodríguez-Jiménez, Víctor Gómez-Mayordomo, Juan Torres-Macho, Oscar J. Pellicer-Valero, José D. Martín-Guerrero, Valentín Hernández-Barrera, Lars Arendt-Nielsen

**Affiliations:** 1 Department of Physical Therapy, Occupational Therapy, Rehabilitation and Physical Medicine, Universidad Rey Juan Carlos, Alcorcón, 28922 Madrid, Spain; 2 Center for Neuroplasticity and Pain (CNAP), SMI, Department of Health Science and Technology, Faculty of Medicine, Aalborg University, 9220 Aalborg, Denmark; 3 Department of Neurology, Hospital Clínico San Carlos, 28040 Madrid, Spain; 4 Department of Internal Medicine, Hospital Universitario Infanta Leonor-Virgen de la Torre, 28031 Madrid, Spain; 5 Department of Medicine, School of Medicine, Universidad Complutense de Madrid, 28040 Madrid, Spain; 6 Intelligent Data Analysis Laboratory, Department of Electronic Engineering, ETSE (Engineering School), Universitat de València (UV), 46010 Valencia, Spain; 7 Department of Public Health, Universidad Rey Juan Carlos (URJC), 28922 Madrid, Spain; 8 Department of Medical Gastroenterology, Mech-Sense, Aalborg University Hospital, 9220 Aalborg, Denmark

**Keywords:** Alpha, Delta, variants, musculoskeletal pain, prevalence

## Abstract

We compared the prevalence of musculoskeletal post-COVID pain between previously hospitalized COVID-19 survivors infected with the historical, Alpha or Delta SARS-CoV-2 variant. Data about musculoskeletal post-COVID pain were systematically collected through a telephone interview involving 201 patients who had survived the historical variant, 211 who had survived the Alpha variant and 202 who had survived the Delta variant six months after hospital discharge. Participants were recruited from non-vaccinated individuals hospitalized due to SARS-CoV-2 infection in one hospital of Madrid (Spain) during three different waves of the pandemic (historical, Alpha or Delta variant). Hospitalization and clinical data were collected from hospital medical records. In addition, anxiety/depressive levels and sleep quality were also assessed. The prevalence of musculoskeletal post-COVID pain was higher (*p* = 0.003) in patients infected with the historical variant (47.7%) than in those infected with the Alpha (38.3%) or Delta (41%) variants. A significantly (*p* = 0.002) higher proportion of individuals infected with the historical variant reported generalized pain (20.5%) when compared with those infected with the other variants. The prevalence of new-onset post-COVID musculoskeletal pain reached 80.1%, 75.2% and 79.5% of patients infected with the historical, Alpha or Delta variants, respectively. No specific risk factors for developing post-COVID pain were identified depending on the SARS-CoV-2 variant. In conclusion, this study found that musculoskeletal post-COVID pain is highly prevalent in COVID-19 survivors six months after hospital discharge, with the highest prevalence and most generalized pain symptoms in individuals infected with the historical variant. Approximately 50% developed “de novo” post-COVID musculoskeletal pain symptoms.

## 1. Introduction

Musculoskeletal pain is a common symptom experienced by almost 20% of people during the acute phase of the severe acute respiratory syndrome coronavirus 2 (SARS-CoV-2), the agent causing the coronavirus disease of 2019 (COVID-19) [1,2]. Additionally, pain is also a common symptom experienced during a post-COVID phase by individuals with long COVID [3]. A meta-analysis reported an overall prevalence of musculoskeletal post-COVID pain of up to 23.6% during the first six months after SARS-CoV-2 infection [4]. This prevalence rate was based on studies investigating general post-COVID symptomatology. In fact, the prevalence of musculoskeletal post-COVID pain raised to rates ranging from 45% to 60% in studies focusing on or specifically investigating this symptom [5,6,7,8,9]. Further, Yelin et al. have defined the so-called “pain-syndrome pattern”, a subgroup of subjects with pain as the main post-COVID symptom [10]. Accordingly, understanding the development of post-COVID pain after SARS-CoV-2 infection seems to be crucial.

After the historical variant discovered in China, several variants such as Alpha, Beta, Gamma, Delta, Epsilon, Zeta, Eta, Theta, Iota, Kappa, Lambda and Omicron have been described [11]. Among all these variants, Alpha (B.1.1.7), Beta (B.1.351), Gamma (p.1), Delta (B.1.617.2) and, more recently, Omicron (B.1.1.529/BA.1) have been considered as variants of concern [12]. A higher transmissibility, a potential immunity to current vaccines and a possibility of reinfections have been different topics of research related to these variants of concern [11,12].

Most studies investigating the presence of musculoskeletal post-COVID pain have primarily included individuals infected with the historical variant [4]. Better understanding the association of post-COVID pain with SARS-CoV-2 variants is warranted. No previous study has systematically compared the presence of musculoskeletal post-COVID pain in individuals infected with different SARS-CoV-2 variants. The main objective of this study was to compare the prevalence of musculoskeletal post-COVID pain between patients hospitalized with one of the three different variants of concern (historical, Alpha or Delta variant) and its clinical features. The secondary aim was to identify possible risk factors associated with the development of musculoskeletal post-COVID pain according to each SARS-CoV-2 variant.

## 2. Methods

### 2.1. Participants

A cross-sectional cohort study including individuals hospitalized due to acute SARS-CoV-2 infection in an urban hospital during three different waves of the COVID-19 epidemic in Madrid, Spain was conducted. All participants should have been diagnosed with SARS-CoV-2 infection with a real-time reverse transcription-polymerase chain reaction (RT -PCR) assay and the presence of clinical or radiological findings at hospitalization. All RT-PCR positive nasopharyngeal/oral samples from those patients hospitalized during the third and fifth waves were screened to identify the SARS-CoV-2 viral lineage. Sanger sequencing of the receptor binding domain (RBD) was used to assess each SARS-CoV-2 variant. All patients included from the first wave (March–April 2020) presented the historical variant, as no other variant was present; all patients from the third wave (January–February 2021) included in the current study should be diagnosed with the Alpha (B.1.1.7) variant; those participants included from the fifth wave (July–August 2021) should present the Delta (B.1.617.2) variant. We only included patients who had not received any dose of vaccination at the time of hospital admission. The study was approved by all the Local Ethics Committees of the Hospital Universitario Infanta Leonor (HUIL/092-20). All participants provided informed consent before their inclusion in the study. 

### 2.2. Data Collection

Specific data focusing on pain symptoms were collected through a telephone semi-structured interview conducted by trained health care researchers. We used the definition of the International Association for the Study of Pain (IASP) of chronic primary musculoskeletal pain for defining post-COVID pain [13]. In addition, symptoms should be present for at least three months and persist at the time of assessment. Participants described the location of their symptoms (e.g., neck, shoulder, elbow, upper extremity, chest, spine, knee, hip, lower extremity or generalized). We did not include head pain due to the particular headache classification and the need for a specific diagnosis according to the classification. Participants with underlying medical conditions that could best explain pain, e.g., arthritis, were excluded.

Hospital medical records were consulted for the obtention of the following data: age, gender, height, weight, previous comorbidities, COVID-19 symptoms at hospital admission, days of hospitalization and intensive care unit (ICU) admission needed.

The Hospital Anxiety and Depression Scale (HADS) was used to evaluate anxiety (HADS-A, 7-items, 0–21 points) and depressive (HADS-D, 7-items, 0–21 points) symptoms [14]. We used the following cut-off scores as indicators of anxiety (HADS-A ≥ 12 points) or depressive (HADS-D ≥ 10 points) levels [15]. The Pittsburgh Sleep Quality Index (PSQI, 0–21 points) was used for evaluating sleep quality during the previous month [16]. We used a cut-off score PSQI ≥ 8.0 points as indicative of being a poor sleeper [16]. Both the HADS and the PSQI can be properly assessed by telephone [17].

### 2.3. Statistical Analysis

Means (standard deviation, SD) are presented for continuous variables, whereas the numbers of cases (percentage) are presented for categorical variables. For the first objective, we compared differences between patients with musculoskeletal post-COVID pain according to the SARS-CoV-2 variant with Chi-squared tests for categorical variables or one-way-ANOVA tests for continuous variables. The level of significance was set at 0.05, with the *p*-values being corrected with the Holm–Bonferroni correction. For the second objective, multivariate logistic regressions were conducted to identify the association of developing musculoskeletal post-COVID pain with variables collected at hospitalization on each SARS-CoV-2 variant. The results of the regressions are presented as an adjusted odds ratio (OR), and their confidence intervals (95%CI) were calculated. The data were collected with the STATA 16.1 program and processed with Python’s library pandas 0.25.3 program. The Scipy 1.5.2 program was used for the statistical analysis, and the Statsmodels 0.11.0 program applied the Holm–Bonferroni correction of the *p*-value.

## 3. Results

Two hundred and fifty (*n* = 250) hospitalized patients from each wave were invited to participate. Finally, 201 (mean age: 60.5, SD: 10.5 years, 54.2% women) individuals from the first wave with the historical variant, 211 (mean age: 70, SD: 15.5 years, 51.1% women) from the third wave with the Alpha variant and 202 (mean age: 56.5, SD: 21.0 years, 54.4% women) from the fifth wave with the Delta variant were included. The patients infected with the Alpha variant were older than those with the historical or Delta variants (*p* < 0.001). All participants were assessed at a similar follow-up period after hospital discharge: historical (mean: 6.5, SD: 1.0 months), Alpha (mean: 6.0, SD: 1.2 months) and Delta (mean: 6.3, SD: 1.0 months) variant.

**Figure 1:**
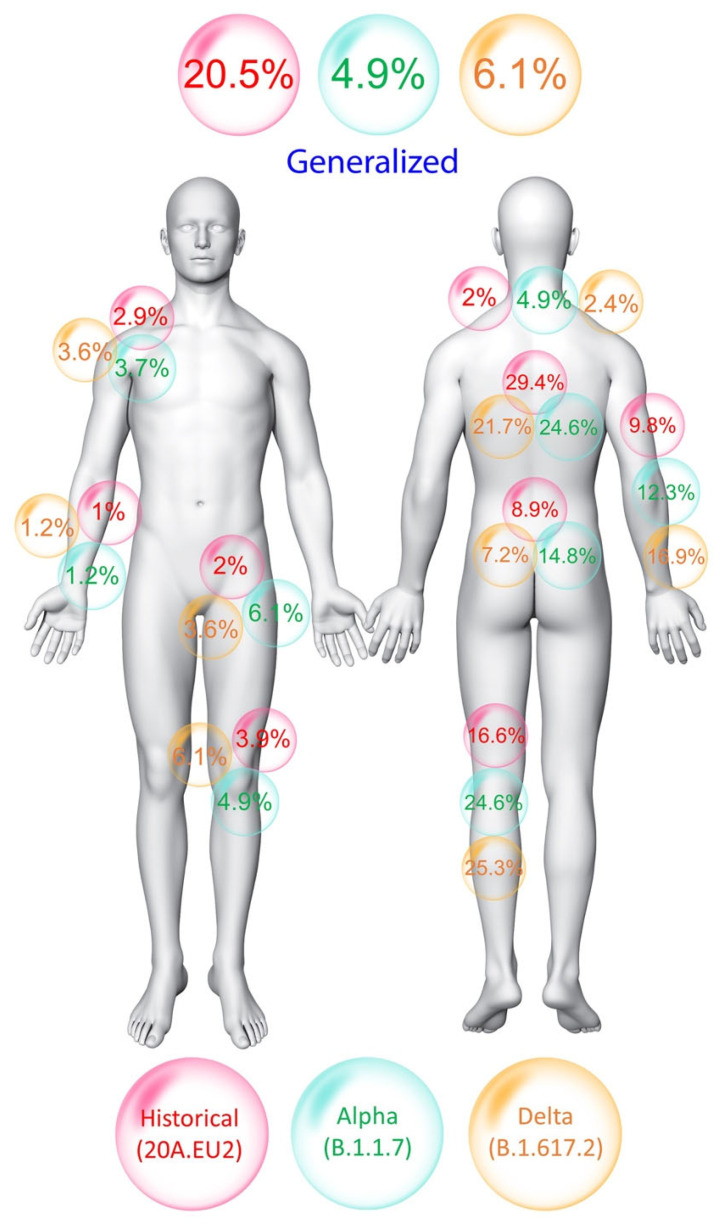
Location of musculoskeletal post-COVID pain symptoms six months after hospital discharge in individuals infected with the historical, Alpha or Delta SARS-CoV-2 variant.

The prevalence of musculoskeletal post-COVID pain six months after hospitalization was significantly higher (*p* = 0.03) in individuals infected with the historical variant (47.7%) when compared with those infected with the Alpha (38.3%, adj OR 1.59, 95%CI 1.06–2.37, *p* = 0.024) or Delta (41%, adj OR 1.50, 95%CI 1.01–2.24, *p* = 0.046) variants. In addition, a significantly (*p* = 0.002) higher proportion of individuals infected with the historical variant reported generalized pain (20.5%) compared to those infected with the other variants. The prevalence of thoracic/chest (29.4%) pain was also higher in the historical variant, although the differences did not reach statistical significance. The prevalence of pain symptoms within the lower and upper extremities was higher in those infected with the Delta variant (25.3% and 16.9%, respectively), although the differences were not significant. The locations of musculoskeletal post-COVID pain by SARS-CoV-2 variant are illustrated in Figure 1.

Clinical and hospitalization data between patients with and without musculoskeletal post-COVID pain by SARS-CoV-2 variant are shown in Table 1. Patients with musculoskeletal post-COVID pain infected with the Alpha variant were older and showed a lower number of onset symptoms at hospital admission and a longer stay of hospitalization (all, *p* < 0.01) than patients who reported musculoskeletal post-COVID pain but were infected with the historical or Delta variant. In addition, subjects with musculoskeletal post-COVID pain who were infected by the historical variant exhibited higher depressive levels than those infected with the Alpha or Delta variants (*p* = 0.001). Anxiety levels (*p* = 0.1) and sleep quality (*p* = 0.448) were not significantly different among the three SARS-CoV-2 variants (Table 1).

No significant differences in the presence of pre-existing musculoskeletal pain symptoms before the infection were observed among the three SARS-CoV-2 variants (*p* = 0.470, Table 1). Accordingly, 51%, 54.3% and 59% of individuals infected with the historical, Alpha or Delta variant developed similar amounts of de novo musculoskeletal post-COVID pain since they did not experience symptoms before hospitalization. Additionally, almost half of the individuals suffering from previous pain symptoms (historical variant: 29.1%, *n* = 28/96; Alpha variant: 20.9%, *n* = 17/81; Delta variant: 20.5%, *n* = 17/83) reported that the post-COVID pain was different from their previous pain symptoms; hence, post-COVID pain was “new-onset”. In summary, the prevalence of new-onset post-COVID musculoskeletal pain six months after hospital discharge reached 80.1%, 75.2% and 79.5% of individuals infected with the historical, Alpha or Delta variant, respectively.

The multivariate analysis did not reveal any significant risk factor (for those investigated in this study) associated with the development of musculoskeletal post-COVID pain symptoms according to a specific SARS-CoV-2 variant.

## 4. Discussion

To the best of the authors’ knowledge, this is the first study to date to investigate the prevalence of musculoskeletal post-COVID pain in previously hospitalized COVID-19 survivors infected with different SARS-CoV-2 variants. Musculoskeletal post-COVID pain six months after hospitalization was present in 48% of patients with the historical variant, 41% of those with the Delta variant and 38% of those with the Alpha variant. Among those developing post-COVID pain, the prevalence of “new-onset” musculoskeletal post-COVID pain was almost 80%, independent of the SARS-CoV-2 variant. The prevalence of musculoskeletal post-COVID pain was higher and more generalized in people infected with the historical variant.

### 4.1. SARS-CoV-2 Variant and Musculoskeletal Post-COVID Pain

Clauw et al. [18] proposed two scenarios related with SARS-CoV-2 infection explaining an expected increase in the incidence of musculoskeletal pain which are supported by our results. The first scenario would be the development of new onset musculoskeletal post-COVID pain in infected individuals, whereas the second one is a worsening of symptoms in people with pain before the infection. Previous evidence reported prevalence rates of musculoskeletal post-COVID pain from 45% to 60% [5,6,7,8,9]. The current study provides evidence supporting the idea that musculoskeletal post-COVID pain symptoms are highly prevalent not only after being infected with the historical variant but also after being infected with the Alpha and Delta variants. Additionally, almost 20% of the patients also exhibited an exacerbation of previous symptomatology independently of the SARS-CoV-2 variant, supporting both hypotheses [18].

Widespread pain, thorax/chest pain and lower extremity pain were the most common areas of musculoskeletal post-COVID pain reported by our patients (Figure 1), which is in agreement with previous studies [7,9]. An interesting finding of the current study is that widespread pain was mainly present in subjects infected with the historical variant and present to a lesser extent in those infected with the Alpha or Delta variant. Widespread pain has been also found as a long-term sequela after Severe Acute Respiratory Syndrome (SARS) infection [19]. The presence of widespread pain is in line with previous studies observing that 60% of COVID-19 survivors report multiple pain sites after hospital discharge [20] and that 30% exhibit common clinical features of fibromyalgia syndrome (widespread pain) [21]. It is important to consider that results from previous studies are based on data from patients infected with the historical variant, supporting that this variant could lead to more widespread pain symptomatology than other variants of concern. Similarly, up to 70% of patients experiencing post-COVID pain also exhibit sensitization-associated symptoms [22]. We also observed that individuals with musculoskeletal post-COVID pain who were infected with the historical variant had higher depressive levels than those infected with the other variants. The presence of widespread pain symptoms as well as other system-associated symptoms such as sleep problems and mood disturbances suggests that musculoskeletal post-COVID pain resembles features of “nociplastic” pain [23], and this could be more pronounced in individuals infected with the historical variant. Future studies characterizing the nature of musculoskeletal post-COVID pain depending on the SARS-CoV-2 variant are now needed.

### 4.2. SARS-CoV-2 Variant and Musculoskeletal Post-COVID Pain Mechanisms

A previous cohort study identified that the female gender, a history of previous musculoskeletal pain symptoms before the infection and the presence of myalgia and headaches as onset symptoms at hospital admission were risk factors associated with musculoskeletal post-COVID pain [9]. We were unable to identify any as significant risk factor depending on the SARS-CoV-2 variant in the current study. It is possible that all risk factors are more related to the presence of musculoskeletal post-COVID pain symptoms themselves but independent of the infected variant. For instance, the female gender is identified as a risk factor associated with the development of more post-COVID symptomatology in general [24]. Similarly, the presence of previous musculoskeletal pain symptoms before the infection, another factor associated with musculoskeletal post-COVID pain development [9], was not different for people developing post-COVID pain but infected with a different SARS-CoV-2 variant.

Emerging evidence suggests that musculoskeletal post-COVID pain is a multifactorial symptom, where factors related to the pathogen (SARS-CoV-2 associated-factors) intersect with the host response (within-individual factors) as well as with external (hospitalization-related) and emotional (COVID-19 outbreak-associated) variables. Accordingly, current evidence supports the idea that musculoskeletal pain is a prevalent post-COVID symptom experienced by people infected with the SARS-CoV-2 virus. Although some differences among the historical, Alpha and Delta variants have been identified in this study, the underlying mechanisms explaining the development of musculoskeletal post-COVID pain may be not associated with any variant. In fact, the current literature supports the idea that SARS-CoV-2 variant differences consist of higher transmissibility, different responses to current vaccines or the possibility of reinfections [11,12], but the molecular and immune responses elicited by the SARS-CoV-2 virus itself seem to be similar between all the variants. We can hypothesize that SARS-CoV-2 infection, independently of the variant, leads to a hyper-excitability of the nervous system throughout different pathways, triggering nociplastic pain responses [25]. It is highly possible that the differences observed between the historical and Alpha/Delta variants are more related to factors surrounding the COVID-19 outbreak of a particular variant. For instance, several COVID-19-associated factors (social alarm, fear of contagion, uncertainty about prognosis, development of post-traumatic stress disorders) capable of promoting the presence of musculoskeletal post-COVID pain [26] were more pronounced during the wave associated with the historical variant due to its association with a worldwide lockdown and the beginning of a “new” pandemic [27,28].

### 4.3. Limitations

Although this is the first study investigating differences in musculoskeletal post-COVID pain in different SARS-CoV-2 variants of concern, our data should be considered in light of potential limitations. First, we collected data from previously hospitalized COVID-19 survivors. Data about musculoskeletal post-COVID pain depending on SARS-CoV-2 variant in non-hospitalized patients are still lacking. In fact, our results should not be used to determine the prevalence rates of musculoskeletal post-COVID pain according to each of the SARS-CoV-2 variants of concern included, since the hospitalization rate with consecutive variants seems to be lower; therefore, population-based studies are needed to determine the real rates of musculoskeletal post-COVID pain for each SARS-CoV-2 variant and not just in the hospitalized population. Similarly, the current data are just based on individuals who have not received any vaccine dose. We do not know if vaccination will also have an influence on the current results. Second, serological biomarkers or COVID-19 severity at hospital admission or follow-up were not collected in our study, which could help to elucidate if these parameters are potential risk factors for musculoskeletal post-COVID pain for each SARS-CoV-2 variant. For instance, we do not know if the need for oxygen (including its parameters, e.g., alveolar-arteriolar oxygen gradient) or pharmacological treatments received during hospitalization could influence the development of musculoskeletal post-COVID pain. Third, the data were collected over telephone, a procedure with a potential bias in population-based survey studies but commonly used in post-COVID research [4]. Fourth, since this was a cross-sectional study, we cannot determine the evolution of musculoskeletal post-COVID pain symptoms, making it difficult to exclusively attribute the presence of symptoms at six months after hospitalization to SARS-CoV-2 infection. Fifth, we were not able to characterize post-COVID pain. Since we focused on musculoskeletal pain symptoms, we do not know if the presence of neuropathic post-COVID pain would be different according to the SARS-CoV-2 variant. Studies classifying the nature of post-COVID pain in individuals infected with different variants of concerns are now needed. Finally, we were not able to collect data from the Omicron (B.1.1.529/BA.1) variant, the dominant variant at this moment.

## 5. Conclusions

The current study found that 40% of previously hospitalized COVID-19 survivors report musculoskeletal post-COVID pain six months after hospitalization, independently of the SARS-CoV-2 variant of concern. The prevalence of musculoskeletal post-COVID pain was higher and more generalized in individuals infected with the historical variant than in those infected with the Alpha or the Delta variant. The prevalence of “new-onset” musculoskeletal post-COVID pain reached almost 80%, independently of the variant, in the cohort of patients reporting post-COVID pain. Depending on the trajectory of musculoskeletal post-COVID pain for the different variants, specific management attention may be given to those patients.

## Figures and Tables

**Table 1:** Clinical and hospitalization data in individuals with musculoskeletal post-COVID pain by SARS-CoV-2 variant.

Variables	Historical (*n* = 96)	Alpha (*n* = 81)	Delta (*n* = 83)
Age, mean (SD), years *	59.0 (15.0)	68.0 (14.0)	53.5 (19.5)
Gender, male/female (%)	39 (40.7%)/55 (57.3%)	35 (43.2%)/46 (56.8%)	34 (40.9%)/49 (59.1%)
Weight, mean (SD), kg	77.5 (13.5)	74.2 (15.0)	79.0 (13.0)
Height, mean (SD), cm	167 (10)	165 (8.5)	166 (9)
Medical co-morbidities			
Hypertension	32 (33.3%)	34 (42.0%)	27 (32.5%)
Diabetes	8 (8.3%)	10 (12.35%)	11 (13.25%)
Cardiovascular Disease	16 (16.7%)	19 (23.45%)	9 (10.85%)
Rheumatological Disease	2 (2.1%)	0 (0.0%)	1 (1.2%)
Asma	8 (8.3%)	4 (4.95%)	12 (14.45%)
COPD	7 (7.3%)	5 (6.2%)	3 (3.6%)
Obesity *	5 (5.2%)	9 (11.1%)	28 (33.7%)
Other (Cancer, Kidney Disease)	13 (13.5%)	30 (37.05%)	18 (21.7%)
Previous Musculoskeletal Pain, n (%)	47 (49%)	37 (45.7%)	34 (41.0%)
Exacerbation Previous Musculoskeletal Pain, n (%)	19 (19.8%)	20 (24.6%)	17 (20.5%)
Number of onset symptoms at hospital admission, mean (SD) *	2.45 (0.7)	2.1 (0.9)	2.6 (0.7)
Onset symptoms at hospital admission, n (%)			
Fever	74 (77.1%)	42 (51.85%)	50 (60.25%)
Dyspnoea	38 (39.6%)	29 (35.8%)	17 (20.5%)
Myalgia	26 (27.1%)	36 (44.45%)	31 (37.35%)
Cough	24 (25.1%)	19 (23.45%)	29 (34.95%)
Headache *	26 (27.1%)	14 (17.3%)	32 (38.55%)
Gastrointestinal Disorders	19 (19.8%)	4 (4.95%)	6 (7.25%)
Anosmia	7 (7.3%)	2 (2.5%)	19 (22.9%)
Ageusia *	5 (5.2%)	2 (2.5%)	14 (16.9%)
Throat Pain	6 (6.25%)	10 (12.35)	14 (16.9%)
Stay at the hospital, mean (SD), days *	15.0 (13.5)	22 (18)	11.5 (12.5)
ICU admission			
Yes/No, n (%)	17 (16.6%)/85 (83.4%)	19 (23.4%)/62 (76.6%)	10 (12%)/73 (88%)
Stay at ICU, mean (SD), days	14.5 (11.5)	18.0 (19.0)	11.0 (6.5)
HADS-D (0–21), mean (SD) *	5.4 (5.0)	3.5 (3.7)	3.9 (3.4)
HADS-D ≥ 10 points, n (%) *	25 (24.5%)	8 (9.8%)	5 (6.0%)
HADS-A (0–21), mean (SD)	4.3 (4.4)	3.0 (3.2)	3.7 (3.3)
HADS-A ≥ 12 points, n (%)	6 (5.8%)	1 (1.25%)	1 (1.2%)
PSQI (0–21), mean (SD)	6.8 (4.2)	7.1 (4.1)	7.5 (3.2)
PSQI ≥ 8 points, n (%)	33 (32.35%)	33 (40.7%)	36 (43.3%)

COPD: Chronic Obstructive Pulmonary Disease; ICU: Intensive Care Unit. * Significant differences between SARS-CoV-2 variants.

## Data Availability

All data relevant to the study are included in the article.
